# Association of WISP1/CCN4 with Risk of Overweight and Gestational Diabetes Mellitus in Chinese Pregnant Women

**DOI:** 10.1155/2020/4934206

**Published:** 2020-04-15

**Authors:** Lei Liu, Jiajin Hu, Liu Yang, Ningning Wang, Yang Liu, Xiaotong Wei, Ming Gao, Yinuo Wang, Yanan Ma, Deliang Wen

**Affiliations:** ^1^Institute of Health Sciences, China Medical University, Shenyang, Liaoning Province 110122, China; ^2^Research Center of China Medical University Birth Cohort, China Medical University, Shenyang, Liaoning Province 110122, China; ^3^Department of Obstetrics and Gynecology, Shenyang Maternity and Child Health Hospital, Shenyang, Liaoning Province 110122, China; ^4^School of Public Health, Dalian Medical University, Dalian, Liaoning Province 116044, China; ^5^School of Public Health, China Medical University, Shenyang, Liaoning Province 110122, China

## Abstract

**Background:**

Obese women with gestational diabetes mellitus (GDM) have a higher risk of adverse outcomes than women with obesity or GDM alone. Our study is aimed at investigating the discriminatory power of circulatory Wnt1-inducible signaling pathway protein-1 (WISP1), a novel adipocytokine, on the copresence of prepregnancy overweight/obesity and GDM and at clarifying the relationship between the WISP1 level and clinical cardiometabolic parameters.

**Methods:**

A total of 313 participants were screened from a multicenter prospective prebirth cohort: Born in Shenyang Cohort Study (BISCS). Subjects were examined with a 2 × 2 factorial design for body mass index (BMI) ≥ 24 and GDM. Between 24 and 28 weeks of pregnancy, follow-up individuals underwent an OGTT and blood sampling for cardiometabolic characterization.

**Results:**

We observed that the WISP1 levels were elevated in prepregnancy overweight/obesity patients with GDM, compared with nonoverweight subjects with normal blood glucose (3.45 ± 0.89 vs. 2.91 ± 0.75 ng/mL). Multilogistic regression analyses after adjustments for potential confounding factors revealed that WISP1 was a strong and independent risk factor for prepregnancy overweight/obesity with GDM (all ORs > 1). In addition, the results of the ROC analysis indicated that WISP1 exhibited the capability to identify individuals with prepregnancy overweight/obesity and GDM (all AUC > 0.5). Finally, univariate and multivariate linear regression showed that WISP1 level was positively and independently correlated with fasting blood glucose, systolic blood pressure, and aspartate aminotransferase and was negatively correlated with HDL-C and complement C1q.

**Conclusions:**

WISP1 may be critical for the prediction, diagnosis, and therapeutic strategies against obesity and GDM in pregnant women.

## 1. Introduction

Globally, the prevalence of obesity is rising to pandemic proportions [1, 2]. More and more women in their childbearing age are overweight or obese [3, 4]. According to a survey in the USA, 55.8% of women of childbearing years (20–39 years) were overweight or obese [5]. In the Chinese population, the rate of maternal overweight and obesity in pregnancy is as high as 25.1% [6]. Gestational diabetes mellitus (GDM) is another serious problem during pregnancy. Globally, GDM affects 3-25% pregnancies, and the continued increase in the incidence of this disease is consistent with an increasing prevalence of obesity [7]. Both maternal obesity and GDM are independent risk factors for obstetric and neonatal complications, such as caesarean section, macrosomia, preeclampsia, or other metabolic disorders at multiple life stages in the offspring [8, 9]. It seems, nonetheless, that obese women with GDM appear to have a higher risk of adverse outcomes than women who suffered from obesity alone or GDM alone [10, 11]. Numerous lines of population-based cohorts and animal studies have emphasized that maternal obesity and GDM are pathological conditions with long-term adverse consequences on cardiovascular metabolism both in mothers and the offspring [9].

Wnt1-inducible signaling pathway protein-1 (WISP1), also known as CCN4, is both an intracellular and a secreted extracellular protein belonging to the CCN protein family and is the target gene of the Wingless-type (Wnt) signaling pathway. Recently, new therapeutic strategies are now focusing upon the role of extracellular matrix-associated proteins such as the proteins of the CCN family [12]. WISP1 is involved in a wide range of biological functions and pathological processes, such as cell growth, differentiation, and survival [13, 14]. And overexpressed WISP1 has been observed in several diseases including GDM [15], hypertension [16], obesity [17, 18], lung fibroblasts [19], and several types of cancer [20]. WISP1 is widely expressed in normal tissues, particularly in human adipose tissue. In addition, WISP1 is also a novel adipokine, secreted by differentiated adipocytes, which stimulates cytokine responses in adipose tissue-associated macrophages and is involved in adipose tissue dysfunction [18]. Of the CCN family members, WISP1 is increasingly recognized as a potential target for the diabetes-related complications [21]. And growing evidence links the Wnt signaling pathway to the modulation of adipogenesis and low-grade inflammation in obesity [18]. These reports provide evidence that WISP1 plays a critical part in the pathogenesis of obesity- and inflammation-related diseases [18]. And previous research revealed that circulatory levels of WISP1 adipokine were higher in obese patients accompanied with increased insulin resistance [22]. Jung et al. demonstrated that WISP1 may play an essential role in obesity-induced hepatic steatosis and insulin resistance [23]. In diabetes research, tissue biopsies showed greater WISP1 expression among diabetic subjects compared with nondiabetic subjects independent of glycemic control in adult males [24]. WISP1 expression has been found to participate in cell and tissue homeostasis through a variety of autocrine and paracrine functions, making it an extremely attractive therapeutic target for medical applications [25]. Further attention needs to focus on WISP1 regulation in vivo to ascertain the therapeutic capacity of WISP1.

Herein, the aims of this study were to evaluate the circulatory WISP1 concentration in pregnant women with overweight/obesity and GDM in a cross-sectional study, to examine the discriminatory power of WISP1 on the copresence of prepregnancy overweight/obesity and GDM, and to clarify the relationship between the WISP1 level and clinical cardiometabolic parameters.

## 2. Materials and Methods

### 2.1. Ethics Statement

This study was approved by the ethics committee of the China Medical University, and informed consent was obtained from all pregnant volunteers.

### 2.2. Study Design and Subjects

Subjects were recruited from a multicenter prospective prebirth cohort: Born in Shenyang Cohort Study (BISCS) as described elsewhere [26]. In the current analysis, a total of 313 singleton pregnant women who met the following eligibility criteria were selected from a pool of 1260 Chinese pregnant women: (1) subjects collected fasting venous blood samples between the 24th and 28th weeks of gestation; (2) women without preexisting diabetes mellitus; (3) subjects without missing or incomplete records of prepregnancy BMI and oral glucose tolerance test (OGTT); and (4) no current regular medications. We compared the demographic parameters of the 313 subjects included in the present study with subjects excluded. We found similar characteristics between the two groups (e.g., age: 29.63 ± 4.19 vs. 30.34 ± 3.85 years, *P* = 0.360; prepregnancy BMI: 22.38 ± 3.96 vs. 22.09 ± 3.58, *P* = 0.140).

The maternal prepregnancy weight (self-reported information) was extracted from the Maternal and Child Health Handbook. The participants' heights were measured under standardized conditions by trained medical examiners at the first prenatal care visit. Prepregnancy BMI was calculated as weight before gestation over height squared. We followed the definition of prepregnancy BMI classification proposed by the Working Group on Obesity in China (WGOC) (underweight: <18.5 kg/m^2^, normal weight: 18.5-23.9 kg/m^2^, overweight: 24.0-27.9 kg/m^2^, and obese: >28.0 kg/m^2^).

GDM refers to various degrees of glucose tolerance abnormalities that occur or are first detected during pregnancy. Between 24 and 28 weeks of gestation, subjects were informed to return to the hospital in their fasting state for antenatal health caring. A 2-hour 75 g oral glucose tolerance test (OGTT) was performed once in follow-up individuals. Venous blood samples were collected at 0 (fasting), 1, and 2 h after a 75 g glucose load. The blood glucose concentrations were detected by a biochemical analyzer (ARCHITECT c1600, Japan). According to the criteria established by the Ministry of Health (MOH) of China, blood glucose threshold values for OGTT at 0 h, 1 h, and 2 h were 5.1, 10.0, and 8.5 mmol/L, respectively. If any measurement met or exceeded these threshold values, the participant was diagnosed with GDM [27].

Based on prepregnancy BMI and OGTT, subjects were divided into four groups: (1) normal weight subjects with normal blood glucose (NO_NBG; *n* = 196), (2) overweight or obesity subjects with normal blood glucose (O_NBG; *n* = 56), (3) normal weight subjects with GDM (NO_GDM; *n* = 33), and (4) overweight or obesity subjects with GDM (O_GDM; *n* = 28).

### 2.3. Blood Sample Collection and Treatment

Elbow venous blood samples were drawn after 12-hour fasting at OGTT (0 h) using blood tubes containing EDTA (Becton Dickinson and Co., UK). The blood samples were immediately centrifuged at 3000 rpm for 10 min at 4°C, and the EDTA-plasma was aliquoted and stored at −80°C until assays.

### 2.4. Clinical and Biochemical Measurements

The plasma total protein, albumin, aspartate aminotransferase (AST), alanine aminotransferase (ALT), prealbumin, total bilirubin, conjugated bilirubin (CB), unconjugated bilirubin (UCB), monoamine oxidase (MAO), total bile acid, urea, creatinine, uric acid, cystatin C, complement C1q, triglyceride, cholesterol, high-density lipoprotein cholesterol (HDL-C), low-density lipoprotein cholesterol (LDL-C), apolipoprotein A1, and apolipoprotein B were detected by an automatic biochemical analyzer (ARCHITECT c1600, Japan), and C-reactive protein (CRP) was analyzed by an automatic special protein analyzer (Beckman Coulter image 800, USA). The hemoglobin and white blood cell (WBC) count was measured using an automatic hematology analyzer (Beckman Coulter DxH 600, USA). The levels of secreted IL-6 were measured using an automatic electrochemiluminescence immunoassay system (Roche Cobas 6000, Switzerland).

WISP1 was detected by monoclonal antibody-based commercial enzyme-linked immunosorbent assay (ELISA) kit (Cloud-Clone Corp., USA) according to the manufacturer's instructions with intra- and interassay CVs < 10% and <12%, respectively. Measurements were performed in duplicates by a single researcher to avoid the investigator bias.

### 2.5. Statistical Analysis

Continuous variables are presented as the mean ± standard deviation (SD), which was compared between the four groups using analysis of variance (ANOVA), followed by post hoc analysis with Bonferroni's correction. Categorical variables are presented as frequencies or percentages, and unadjusted comparisons among four groups were performed for significance using *χ*^2^ tests. The general linear model was adopted for calculating interaction between prepregnancy overweight/obesity and GDM as independent factors with binary traits on circulatory WISP1 levels. A multilogistic regression analysis after adjustments for potential confounding factors (models 1, 2, 3, 4, and 5) was performed to identify the relationship between WISP1 and prepregnancy overweight/obesity with GDM. Odds ratios (ORs) and 95% confidence intervals (CIs) were reported. And the area under the receiver operating characteristic (ROC) curve (AUC) was measured to observe the discriminatory performance of WISP1 for the copresence of prepregnancy overweight/obesity and GDM risk. Additionally, univariate and multivariate regression analyses were carried out to identify independent variables of WISP1. Statistical significance was defined as *P* < 0.05. All statistical analyses were performed using SPSS 20.0.

## 3. Results

### 3.1. Findings in the Cross-Sectional Study

A total of 313 participants were enrolled into the study, including 196 with normal prepregnancy weight and blood glucose, 56 with prepregnancy overweight/obesity and normal blood glucose, 33 with normal prepregnancy weight and GDM, and 28 with prepregnancy overweight/obesity and GDM. Demographic characteristics and laboratory data of the study subjects are shown in Tables 1 and 2. Maternal gestational age, ethnicity, gravidity, height, and hemoglobin were not statistically different among four groups. Pregnant women with GDM showed significantly higher age. Meanwhile, women who were prepregnancy overweight/obesity (O_NBG and O_GDM groups) tended to have higher systolic and diastolic BP (*P* < 0.001).

Table 2 shows the features of the clinical cardiometabolic profiles of the four categories of participants. In terms of inflammatory markers, the O_GDM and O_NBG groups had a higher WBC count and circulating CRP concentration than the NO_NBG group. Regarding liver and kidney function markers, plasma levels of ALT, AST, urea, and creatinine exhibited no differences among four groups. Furthermore, the circulating triglyceride was significantly higher in the O_GDM group than in the NO_NBG group, but the levels of total cholesterol, HDL-C, LDL-C, and apolipoprotein A1 and B have no significant differences among the four groups.

### 3.2. Comparison of WISP1 among the Four Groups

**Figure 1:**
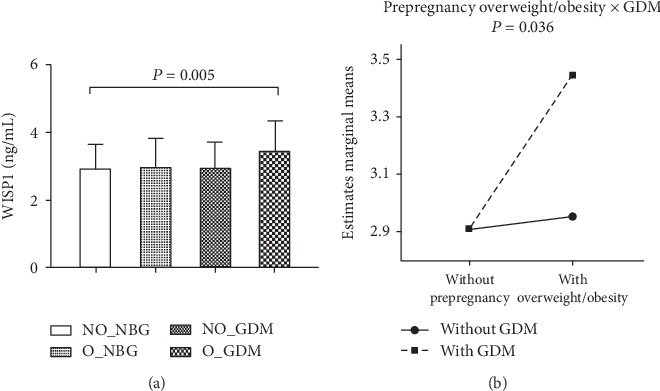
Comparison of circulatory abundance of WISP1 among the four groups. (a) Bar graphs represent plasma WISP1 concentration, and (b) line graphs represent the directions of interaction effect of obesity and GDM on WISP1 in pregnant women categorized into four groups, including (1) nonoverweight or obesity subjects with normal blood glucose (NO_NBG; *n* = 196), (2) overweight or obesity subjects with normal blood glucose (O_NBG; *n* = 56), (3) nonoverweight or obesity subjects with gestational diabetes mellitus (NO_GDM; *n* = 33), and (4) overweight or obesity subjects with gestational diabetes mellitus (O_GDM; *n* = 28).

In the total participants, the mean concentration of plasma WISP1 was 2.97 ± 0.80 ng/mL. As shown in Figure 1(a), the circulating level of WISP1 in the O_GDM subjects was 19% higher than that in the NO_NBG group (*P* = 0.005), but there exhibited no significant changes in the O_NBG and NO_GDM groups compared with the NO_NBG group. Furthermore, through 2 × 2 factorial design analysis, we found an interaction effect between prepregnancy overweight/obesity and GDM in circulatory WISP1 concentration (*P* = 0.036; Figure 1(b)).

### 3.3. Multilogistic Regression Analysis to Identify the Association between the Concentration of WISP1 and the Presence or Absence of Prepregnancy Overweight/Obesity and GDM

**Figure 2:**
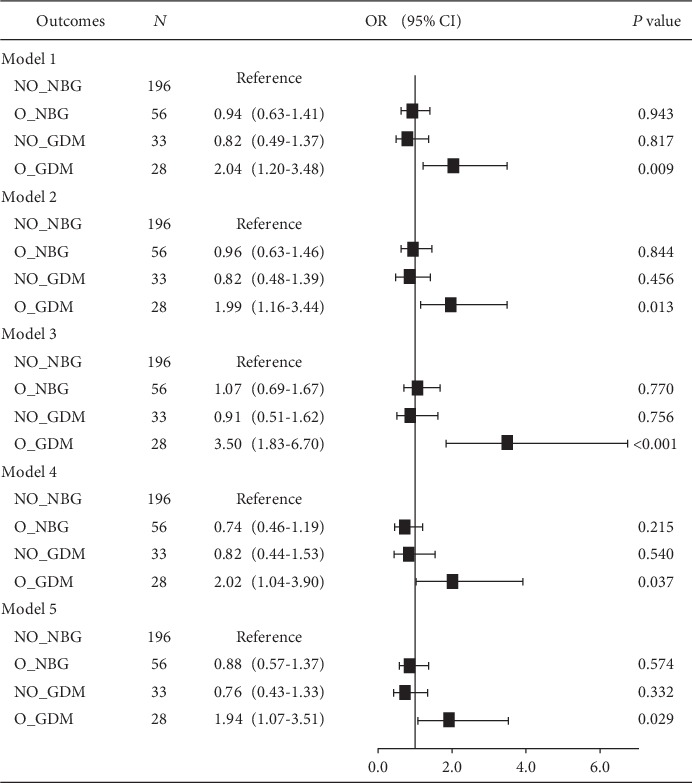
Multilogistic regression analysis to determine the relationship between WISP1 and the presence or absence of prepregnancy overweight and GDM. Model 1: adjusted for age, gestational age, SBP, and DBP. Model 2: adjusted for age, gestational age, SBP, DBP, WBC, IL-6, and CRP. Model 3: adjusted for age, gestational age, SBP, DBP, ALT, and AST. Model 4: adjusted for age, gestational age, SBP, DBP, urea, creatinine, uric acid, cystatin C, and complement C1q. Model 5: adjusted for age, gestational age, SBP, DBP, triglyceride, cholesterol, HDL-C, and LDL-C.

Multilogistic regression analyses after adjustments for age, gestational age, SBP, and DBP (model 1); after adjustments for age, gestational age, SBP, DBP, WBC, IL-6, and CRP (model 2); after adjustments for age, gestational age, SBP, DBP, ALT, and AST (model 3); after adjustments for age, gestational age, SBP, DBP, urea, creatinine, uric acid, cystatin C, and complement C1q (model 4); and after adjustments for age, gestational age, SBP, DBP, triglyceride, cholesterol, HDL-C, and LDL-C (model 5) were performed to evaluate the association between WISP1 concentration and the prevalence of prepregnancy overweight/obesity or GDM. Adjusted analysis of potential confounders disclosed that the O_GDM group was the only group exhibiting a statistical significance in the overall models (Figure 2). Using the multilogistic regression analysis, we revealed that WISP1 was a strong independent risk predictor of comorbidity of prepregnancy overweight/obesity and GDM (model 1: OR = 2.04, 95% CI 1.20-3.48; model 2: OR = 1.99, 95% CI 1.16-3.44; model 3: OR = 3.50, 95% CI 1.83-6.70; model 4: OR = 2.02, 95% CI 1.04-3.90; and model 5: OR = 1.94, 95% CI 1.07-3.51). In brief, WISP1 had a stronger relationship with the copresence of both diseases than with either alone.

### 3.4. Receiver Operating Characteristic Analysis of Circulating WISP1 Levels for Identifying Prepregnancy Overweight/Obesity Subjects with GDM

**Figure 3:**
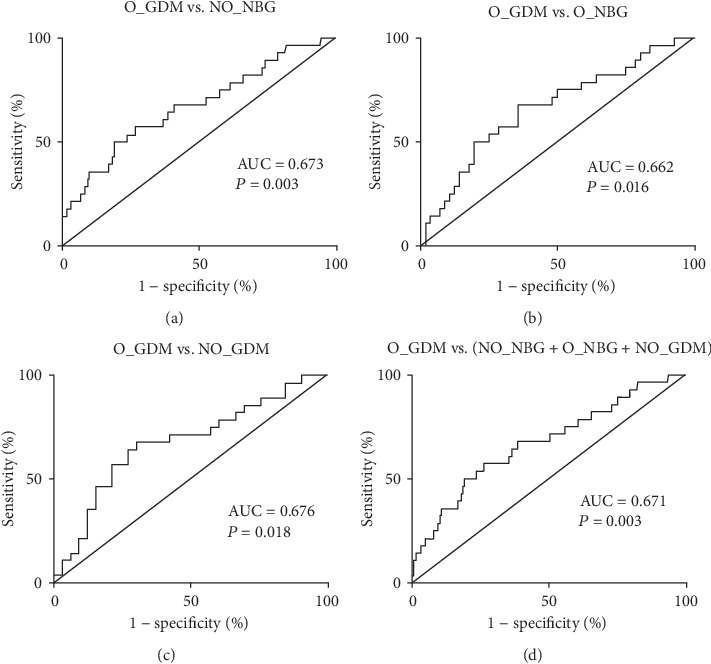
Receiver operating characteristic analysis of intercept and slope of circulating WISP1 levels for distinguishing prepregnancy overweight/obesity subjects with GDM. ROC curve analysis was carried out for O_GDM versus (a) NO_NBG, (b) O_NBG, and (c) NO_GDM groups separately and (d) total population to find how WISP1 performed to predict prepregnancy overweight/obesity and GDM.

To evaluate the predictive effect of WISP1 on prepregnancy overweight/obesity and GDM, ROC curve analysis was carried out for the O_GDM versus NO_NBG, O_NBG, and NO_GDM groups separately and combined group (Figures 3(a)–3(d)). Between the O_GDM versus NO_NBG groups, the area under the ROC curve was 0.673 (95% CI: 0.560-0.787); between the O_GDM versus O_NBG groups, it was 0.662 (95% CI: 0.537-0.787); between the O_GDM versus NO_GDM groups, it was 0.676 (95% CI: 0.539-0.814); and between the O_GDM versus combined groups, it was 0.671 (95% CI: 0.561-0.782). Thus, WISP1 exhibited satisfactory capacity to discriminate the copresence of prepregnancy overweight/obesity and GDM.

### 3.5. Univariate and Multivariate Regression Analyses of Variable WISP1

As presented in Table 3, among all demographic and clinical cardiometabolic determinants, bivariate correlation analyses showed that WISP1 levels were positively associated with weight (*r* = 0.136, *P* = 0.016), BMI (*r* = 0.124, *P* = 0.028), FBG (*r* = 0.168, *P* = 0.003), SBP (*r* = 0.169, *P* = 0.003), circulating CRP concentrations (*r* = 0.135, *P* = 0.017), total protein (*r* = 0.461, *P* < 0.001), albumin (*r* = 0.417, *P* < 0.001), AST (*r* = 0.247, *P* < 0.001), prealbumin (*r* = 0.325, *P* < 0.001), CB (*r* = 0.176, *P* = 0.002), UCB (*r* = 0.189, *P* = 0.001), total bile acid (*r* = 0.227, *P* < 0.001), MAO (*r* = 0.290, *P* < 0.001), urea (*r* = 0.226, *P* < 0.001), uric acid (*r* = 0.365, *P* < 0.001), cystatin C (*r* = 0.498, *P* < 0.001), complement C1q (*r* = 0.272, *P* < 0.001), triglyceride (*r* = 0.251, *P* < 0.001), cholesterol (*r* = 0.288, *P* < 0.001), HDL-C (*r* = 0.157, *P* = 0.005), LDL-C (*r* = 0.242, *P* < 0.001), apolipoprotein A1 (*r* = 0.362, *P* < 0.001), and apolipoprotein B (*r* = 0.361, *P* < 0.001).

All of the variables that correlated with WISP1 at *P* < 0.05 in the bivariate correlation analysis were entered into the multivariate linear regression analysis. The analyses demonstrated that FBG (*β* = 0.318, 95% CI: 0.133-0.603), SBP (*β* = 0.007, 95% CI: 0.000-0.013), AST (*β* = 0.016, 95% CI: 0.002-0.030), UCB (*β* = 0.103, 95% CI: 0.001-0.205), cystatin C (*β* = 1.426, 95% CI: 0.659-2.193), complement C1q (*β* = −0.006, 95% CI: -0.009–-0.002), and HDL-C (*β* = −0.582, 95% CI: -1.097–-0.067) independently predicted WISP1 levels (Table 3).

## 4. Discussion

In the present study, we observed that the WISP1 levels elevated in prepregnancy overweight/obesity with GDM patients, compared with normal weight and blood glucose subjects; and WISP1 was a strong independent risk predictor of the copresence of prepregnancy overweight/obesity and GDM. The data on the role of WISP1 in metabolic disturbances, especially among pregnant women, are very limited. This is the first time to explore the discriminatory power of WISP1 on the copresence of prepregnancy overweight/obesity and GDM and the relationship between WISP1 levels and clinical cardiometabolic parameters in pregnant women with overweight/obesity and GDM in a cross-sectional study.

Wingless-type mouse mammary tumor virus (MMTV) integration site family member 1 (Wnt1) belongs to a family of cysteine-rich, glycosylated signaling proteins that mediate numerous developmental processes, such as modulation of cell proliferation, adhesion, polarity, and fate [28]. WISP1 has recently been recognized as a novel adipokine, a biologically active polypeptide secreted by adipocytes and adipose tissue immune cells [29]. Adipokines have been proposed as the link between obesity and GDM, which is related to the pathogenesis of metabolic diseases [30]. Our results indicated that the plasma concentration of WISP1 was higher in prepregnancy overweight/obesity women with GDM than healthy and overweight or GDM alone women. Two previous studies examined the association between circulating WISP1 and adult obesity status. One showed that WISP1 was substantially overexpressed in visceral fat from obese subjects and reflected insulin resistance and inflammation of adipose tissue [18], whereas another study found that WISP1 levels increased in obese persons and were directly associated with adiposity, independent of glycemic status or insulin resistance [17]. In contrast, our findings demonstrated that prepregnancy overweight/obesity interacted with GDM to affect circulatory concentration of WISP1 (Figure 1(b)). Similarly, it has been reported that circulating WISP1 in the GDM group is significantly higher than that in the control group [15].

Multilogistic regression analyses revealed that WISP1 was a strong and independent risk factor for prepregnancy overweight/obesity combined with GDM (all ORs > 1). In addition, the results of the ROC analysis indicated that WISP1 exhibited the capability to identify individuals with prepregnancy overweight/obesity and GDM (all AUC > 0.5). Taken together, our results collectively suggested that the circulatory WISP1 is a good clinical index for prediction and diagnosis in pregnant women. Previous studies have shown that WISP1 regulates the posttranslational phosphorylation of AMP-activated protein kinase (AMPK), which is involved in glucose homeostasis, adipocyte differentiation, and lipid accumulation [31–34]. Moreover, WISP1 also phosphorylates and activates mammalian target of rapamycin (mTOR) [12, 35, 36] and silent mating type information regulation 2 homolog 1 (SIRT1) [21, 37], molecules that play a vital role in stem cell regulation, programmed cell death, and cellular energy homeostasis [38]. As a potential endogenous reparative response to injury, WISP1 may further provide novel therapies for restoring pancreatic function and controlling lipid metabolism [39].

Furthermore, WISP1 levels were positively and independently correlated with FBG, SBP, AST, UCB, and cystatin C and negatively correlated with HDL-C and complement C1q. In line with our findings, Sahin Ersoy et al. observed that WISP1 was positively correlated with FBG, HOMA-IR values, BMI, fasting insulin, and triglyceride levels [15]. A study from Japan showed that the 2364 A-G polymorphism of WISP1 was correlated with both the prevalence of hypertension and BP in men [16]. Of note, WISP1 expression correlated negatively and independently with circulating HDL-C levels, suggesting that WISP1 may be a useful marker for lipid transport and metabolism. HDL-C is the major component of metabolic syndrome [40] and cardiovascular risk prediction, which is inversely associated with risks of coronary heart disease [41]. Moreover, our study found that WISP1 was positively correlated with CRP (*r* = 0.135, *P* = 0.017), indicating its correlation with inflammatory state. Murahovschi et al. have focused on the relationship between WISP1 and obesity-related inflammatory response [18]. In their in-depth study, a positive correlation between WISP1 mRNA levels and macrophage infiltration was detected. Further, with in vitro experiments, they displayed that stimulation of human macrophages with WISP1 led to a proinflammatory response (e.g., increased mRNA expression of IL-6, TNF*α*, IL-1*β*, and IL-10). Hence, WISP1 has a complex relationship pattern with various cellular pathways in promoting cell survival, tissue restoration, and inflammatory modulation [12].

There are several limitations in the present study. First, due to the cross-sectional data, the exactly causal nexus between WISP1 and risks of prepregnancy overweight/obesity with GDM cannot be inferred. Further studies with longitudinal designs are needed to discuss causal relationships. Second, fewer subjects have the copresence of prepregnancy overweight and GDM. Third, we do not collect data on the weight gain during the pregnancy period, which may affect the adipose tissue mass and therewith the levels of adipokines. Another, all enrolled pregnant women belonged to a single ethnic birth cohort and they were registered in northeast China. Therefore, our conclusion may not be representative of the general pregnant women population.

Maternal obesity and GDM exposure increases the probability of the development of severe adverse perinatal outcomes and metabolic diseases at the later stage of lifetime of the offspring [42], and a better understanding of the pathophysiology and molecular basis of this condition may lead to new therapeutic perspective. Fortunately, WISP1 may be critical for the development of therapeutic strategies against obesity and GDM in pregnant women. This may be a target for further studies aimed at probing the longitudinal changes of this molecule during pregnancy.

## 5. Conclusions

In summary, the WISP1 levels were elevated in prepregnancy overweight/obesity with GDM patients, compared with normal weight and blood glucose subjects; and WISP1 is a strong independent risk predictor for the copresence of prepregnancy overweight/obesity and GDM. WISP1 may be a novel therapeutic target for maternal obesity with GDM.

## Figures and Tables

**Table 1:** Demographic characteristics of study subjects.

Variables	All cases (*n* = 313)	NO_NBG (*n* = 196)	O_NBG (*n* = 56)	NO_GDM (*n* = 33)	O_GDM (*n* = 28)	*P* value
Age (years)	29.63 ± 4.19	29.05 ± 3.91	29.50 ± 4.16	32.67 ± 4.46	30.43 ± 4.37	<0.001
Gestational age (weeks)	24.34 ± 1.32	24.33 ± 1.26	24.29 ± 1.37	24.06 ± 1.78	24.87 ± 0.80	0.109
*Ethnicity*						
Han, *n* (%)	261 (83.4)	162 (82.7)	48 (85.7)	28 (84.8)	23 (82.1)	0.943
Others, *n* (%)	52 (16.6)	34 (17.3)	8 (14.3)	5 (15.2)	5 (17.9)	
*Gravidity*						
1, *n* (%)	156 (49.8)	102 (52.1)	29 (51.8)	14 (42.4)	11 (39.3)	0.711
2, *n* (%)	84 (26.8)	52 (26.5)	15 (26.8)	8 (24.3)	9 (32.1)	
≥3, *n* (%)	73 (23.4)	42 (21.4)	12 (21.4)	11 (33.3)	8 (28.6)	
Height (cm)	162.65 ± 4.92	162.63 ± 4.92	162.64 ± 5.30	162.67 ± 4.83	162.82 ± 4.40	0.998
Weight (kg)	59.27 ± 11.09	54.16 ± 6.06	73.09 ± 9.62	54.58 ± 6.82	72.93 ± 10.40	<0.001
BMI (kg/m^2^)	22.38 ± 3.96	20.46 ± 1.97	27.62 ± 3.21	20.60 ± 2.30	27.47 ± 3.44	<0.001
FBG (mmol/L)	4.56 ± 0.44	4.43 ± 0.31	4.48 ± 0.35	4.84 ± 0.50	5.31 ± 0.48	<0.001
OGTT 1 h (mmol/L)	7.52 ± 1.66	7.02 ± 1.43	7.34 ± 1.23	9.33 ± 1.32	9.22 ± 1.74	<0.001
OGTT 2 h (mmol/L)	6.90 ± 1.29	6.50 ± 0.97	6.62 ± 0.89	8.16 ± 1.23	8.79 ± 1.47	<0.001
SBP (mmHg)	106.50 ± 12.9	103.20 ± 11.3	113.00 ± 13.2	108.42 ± 13.2	114.39 ± 14.2	<0.001
DBP (mmHg)	68.68 ± 8.8	66.61 ± 7.7	71.96 ± 9.2	69.00 ± 7.8	76.21 ± 10.9	<0.001
Hemoglobin (g/L)	126.75 ± 13.3	126.13 ± 13.8	126.29 ± 11.7	126.79 ± 12.4	132.00 ± 13.4	0.184

BMI: body mass index; FBG: fasting blood glucose; OGTT: oral glucose tolerance test; SBP: systolic pressure; DBP: diastolic pressure.

**Table 2:** Clinical cardiometabolic parameters of study subjects.

Variables	All cases (*n* = 313)	NO_NBG (*n* = 196)	O_NBG (*n* = 56)	NO_GDM (*n* = 33)	O_GDM (*n* = 28)	*P* value
WISP1 (ng/mL)	2.97 ± 0.80	2.91 ± 0.75	2.96 ± 0.87	2.91 ± 0.82	3.45 ± 0.89	0.010
*Inflammatory marker*						
WBC count (×10^9^/L)	8.59 ± 6.24	8.04 ± 1.83	11.19 ± 14.01	7.56 ± 1.77	8.52 ± 2.03	0.006
IL-6 (pg/mL)	2.27 ± 0.89	2.15 ± 0.72	2.51 ± 1.32	2.53 ± 0.92	2.36 ± 0.72	0.012
CRP (mg/L)	4.78 ± 3.12	4.14 ± 3.00	6.56 ± 3.24	4.57 ± 2.30	5.97 ± 3.12	<0.001
*Liver function marker*						
Total protein (g/L)	52.57 ± 10.30	52.00 ± 10.59	55.10 ± 9.27	51.98 ± 10.76	52.21 ± 9.38	0.251
Albumin (g/L)	29.37 ± 6.03	29.10 ± 6.35	30.59 ± 5.23	28.90 ± 5.94	29.36 ± 5.30	0.413
Albumin/globulin	1.29 ± 0.14	1.30 ± 0.13	1.25 ± 0.14	1.27 ± 0.15	1.33 ± 0.19	0.070
ALT (U/L)	10.27 ± 7.49	10.52 ± 7.91	9.70 ± 8.02	11.70 ± 6.55	7.96 ± 2.19	0.223
AST (U/L)	12.35 ± 6.38	12.87 ± 6.64	11.66 ± 6.51	12.45 ± 5.67	9.89 ± 4.31	0.105
Prealbumin (g/L)	0.18 ± 0.06	0.18 ± 0.05	0.18 ± 0.04	0.17 ± 0.04	0.21 ± 0.13	0.019
Total bilirubin (*μ*mol/L)	4.44 ± 1.68	4.58 ± 1.76	4.21 ± 1.57	4.55 ± 1.69	3.78 ± 1.12	0.072
CB (*μ*mol/L)	2.64 ± 0.66	2.65 ± 0.66	2.71 ± 0.68	2.62 ± 0.71	2.48 ± 0.56	0.507
UCB (*μ*mol/L)	1.80 ± 1.15	1.93 ± 1.21	1.50 ± 0.99	1.93 ± 1.13	1.30 ± 0.81	0.007
Total bile acid (*μ*mol/L)	1.79 ± 1.27	1.68 ± 1.09	2.11 ± 1.83	2.01 ± 1.23	1.69 ± 1.10	0.104
MAO (U/L)	7.34 ± 2.42	7.24 ± 2.38	8.12 ± 2.71	6.78 ± 2.36	7.21 ± 1.96	0.047
*Renal function marker*						
Urea (mmol/L)	2.23 ± 0.58	2.23 ± 0.55	2.14 ± 0.49	2.37 ± 0.72	2.29 ± 0.74	0.310
Creatinine (*μ*mol/L)	37.28 ± 17.88	37.41 ± 22.04	37.44 ± 5.76	35.80 ± 7.06	37.79 ± 7.55	0.967
Uric acid (*μ*mol/L)	198.5 ± 57.4	192.8 ± 58.5	214.7 ± 56.7	186.6 ± 37.1	220.5 ± 61.9	0.007
Cystatin C (mg/L)	0.68 ± 0.17	0.66 ± 0.17	0.73 ± 0.16	0.67 ± 0.17	0.71 ± 0.18	0.015
Complement C1q (mg/L)	158.3 ± 36.9	155.3 ± 38.0	170.0 ± 33.4	156.1 ± 35.4	158.9 ± 33.8	0.067
*Lipids*						
Triglyceride (mmol/L)	1.92 ± 0.99	1.73 ± 0.72	2.24 ± 1.12	2.12 ± 1.57	2.38 ± 1.20	<0.001
Cholesterol (mmol/L)	4.47 ± 1.98	4.41 ± 1.10	4.34 ± 1.06	4.38 ± 1.17	5.25 ± 5.67	0.185
HDL-C (mmol/L)	1.42 ± 0.43	1.47 ± 0.44	1.33 ± 0.33	1.41 ± 0.42	1.28 ± 0.45	0.051
LDL-C (mmol/L)	2.35 ± 0.77	2.41 ± 0.78	2.27 ± 0.72	2.27 ± 0.72	2.15 ± 0.74	0.292
Apolipoprotein A1 (g/L)	1.59 ± 0.42	1.59 ± 0.44	1.63 ± 0.38	1.61 ± 0.40	1.55 ± 0.44	0.840
Apolipoprotein B (g/L)	0.81 ± 0.24	0.81 ± 0.24	0.84 ± 0.25	0.80 ± 0.27	0.80 ± 0.26	0.889

WBC: white blood cell; IL-6: interleukin-6; CRP: C-reactive protein; ALT: alanine aminotransferase; AST: aspartate aminotransferase; CB: conjugated bilirubin; UCB: unconjugated bilirubin; MAO: monoamine oxidase; HDL-C: high-density lipoprotein cholesterol; LDL-C: low-density lipoprotein cholesterol.

**Table 3:** Univariate and multivariate regression analyses of WISP1.

Variables	Univariate		Multivariate	
	*r*	*P* value	*β* (95% CI)	*P* value
Age (years)	0.080	0.160	—	—
Gestational age (weeks)	0.109	0.055	—	—
Height (cm)	0.050	0.378	—	—
Weight (kg)	0.136	**0.016**	0.003 (-0.018, 0.025)	0.752
BMI (kg/m^2^)	0.124	**0.028**	-0.018 (-0.077, 0.041)	0.550
FBG (mmol/L)	0.168	**0.003**	0.318 (0.133, 0.603)	**0.001**
OGTT 1 h (mmol/L)	0.091	0.108	—	—
OGTT 2 h (mmol/L)	0.044	0.439	—	—
SBP (mmHg)	0.169	**0.003**	0.007 (0.000, 0.013)	**0.044**
DBP (mmHg)	0.085	0.132	—	—
Hemoglobin (g/L)	-0.080	0.158	—	—
*Inflammatory marker*				
WBC count (×10^9^/L)	-0.063	0.263	—	—
IL-6 (pg/mL)	-0.041	0.471	—	—
CRP (mg/L)	0.135	**0.017**	0.025 (-0.002, 0.052)	0.065
*Liver function marker*				
Total protein (g/L)	0.461	**<0.001**	0.025 (-0.002, 0.053)	0.074
Albumin (g/L)	0.417	**<0.001**	0.005 (-0.026, 0.037)	0.750
Albumin/globulin	0.013	0.816	—	—
ALT (U/L)	0.087	0.123	—	—
AST (U/L)	0.247	**<0.001**	0.016 (0.002, 0.030)	**0.028**
Prealbumin (g/L)	0.325	**<0.001**	-0.848 (-2.605, 0.909)	0.343
Total bilirubin (*μ*mol/L)	0.198	0.055	—	—
CB (*μ*mol/L)	0.176	**0.002**	-0.172 (-0.354, 0.011)	0.065
UCB (*μ*mol/L)	0.189	**0.001**	0.103 (0.001, 0.205)	**0.048**
Total bile acid (*μ*mol/L)	0.227	**<0.001**	0.032 (-0.036, 0.101)	0.927
MAO (U/L)	0.290	**<0.001**	-0.019 (-0.063, 0.025)	0.404
*Renal function marker*				
Urea (mmol/L)	0.226	**<0.001**	0.043 (-0.104, 0.190)	0.564
Creatinine (*μ*mol/L)	0.108	0.057	—	—
Uric acid (*μ*mol/L)	0.365	**<0.001**	0.001 (-0.001, 0.003)	0.322
Cystatin C (mg/L)	0.498	**<0.001**	1.426 (0.659, 2.193)	**<0.001**
Complement C1q (mg/L)	0.272	**<0.001**	-0.006 (-0.009, -0.002)	**0.001**
*Lipids*				
Triglyceride (mmol/L)	0.251	**<0.001**	-0.138 (-0.287, 0.010)	0.068
Cholesterol (mmol/L)	0.288	**<0.001**	0.037 (-0.007, 0.080)	0.102
HDL-C (mmol/L)	0.157	**0.005**	-0.582 (-1.097, -0.067)	**0.027**
LDL-C (mmol/L)	0.242	**<0.001**	-0.267 (-0.659, 0.125)	0.182
Apolipoprotein A1 (g/L)	0.362	**<0.001**	0.299 (-0.361, 0.959)	0.373
Apolipoprotein B (g/L)	0.361	**<0.001**	1.180 (-0.188, 2.548)	0.091

## Data Availability

The data used to support the findings of this study are available from the corresponding author upon request.
